# Sense of Coherence and Stress-Related Resilience: Investigating the Mediating and Moderating Mechanisms in the Development of Resilience Following Stress or Adversity

**DOI:** 10.3389/fpsyt.2018.00378

**Published:** 2018-08-21

**Authors:** Shauna L. Mc Gee, Jan Höltge, Andreas Maercker, Myriam V. Thoma

**Affiliations:** ^1^Psychopathology and Clinical Intervention, Institute of Psychology, University of Zürich, Zurich, Switzerland; ^2^University Research Priority Program ‘Dynamics of Healthy Aging', University of Zürich, Zurich, Switzerland

**Keywords:** sense of coherence-revised, stress-related resilience, resilience mechanisms, psychological health, mediating factor

## Abstract

**Background:** Trauma, stress, and adversity are well-known for having lasting negative effects on health. Yet, not all individuals go on to develop psychopathology or impaired health. However, little is known about the underlying mechanisms which influence the development of stress-related resilience. Sense of coherence-revised (SOC-R) may play a role in this process, as it is formed through overcoming stress or adversity. It may also influence the steeling effect, which suggests that previous exposure to moderate adversity increases resilience to later adversities.

**Objectives:** This study aimed to examine the mediating and moderating roles of SOC-R in the relationship between stress or adversity, and psychological health and well-being. It further aimed to investigate the role of SOC-R in steeling processes.

**Methods:** The study used a longitudinal design, with data collection at baseline and one-year follow-up. Participants included (*N* = 238) Swiss older adults (*M*_age_ = 68.3 years). Standardized questionnaires assessed early-life adversity, recent chronic stress, SOC-R, and current health and well-being. Mediation and moderation analyses examined the mechanisms underpinning stress-related resilience and curvilinear associations assessed steeling.

**Results:** Results showed that the Manageability subscale of SOC-R significantly moderated the relationship between chronic stress and general mental health (*b* = 0.04, 95% CI [0.007, 0.082], *t* = 2.32, *p* < 0.05). Furthermore, SOC-R significantly mediated the relationship for general mental health (GMH) and satisfaction with life (SWL) with childhood emotional neglect (GMH: *b* = −0.056, 95% BCa CI [−0.126, −0.002]; SWL: *b* = −0.043, 95% BCa CI [−0.088, −0.004]), childhood physical neglect (GMH: *b* = −0.100, 95% BCa CI [−0.232, −0.002]; SWL: *b* = −0.081, 95% BCa CI [−0.181, −0.002]), and chronic stress (GMH: *b* = −0.052, 95% BCa CI [−0.100, −0.001]; SWL: *b* = −0.055, 95% BCa CI [−0.097, −0.020]). No curvilinear associations were observed between stress or adversity and SOC-R.

**Conclusions:** This study expands on the limited research on stress-related resilience by examining the role of SOC-R in the interactions between adversity, stress, and health. Future research should examine SOC-R in samples with a greater range and different types of adversity. Overall, findings suggest that SOC-R is an important mechanism underpinning the development of stress-related resilience.

## Introduction

Trauma, adversity, and stress exposure can have lasting negative effects on psychological health and well-being. In particular, early-life adversity (such as trauma, maltreatment, or neglect) has been shown to lead to long-term health-related problems and the development of mental health disorders (1, 2). For instance, a large-scale study by the World Health Organization (WHO) examined the prevalence of mental health disorders across 21 countries. Findings revealed that 38.8% of participants had experienced some form of early-life adversity, such as maltreatment and neglect, family violence, or physical abuse. It further revealed that such experiences of early-life adversity accounted for 29.8% of psychological disorders in adulthood (3). In addition, stress exposure is also associated with long-term negative outcomes, with chronic stress in particular being detrimental to health. Continuous or repeated exposure to stress can result in cumulative adverse effects on physiological health (4, 5). This “wear and tear” on the body has been shown to lead to an increased susceptibility to the development of stress-related mental health problems, such as depression, anxiety, and post-traumatic stress disorder (6, 7). However, while stress or adversity can lead to long-term negative outcomes, not all individuals go on to develop psychopathology or impaired physical health.

Many individuals are able to adapt to stress or adversity and maintain good health and quality of life (8, 9). Such heterogeneity in response to stress or adversity can be explained by the concept of “resilience.” Resilience refers to the ability to adapt to experiences of stress or adversity and maintain a stable trajectory of healthy psychosocial and physical functioning (10). For example, with regard to chronic stress, a study by Sharpley et al. (11) examined the relationship between chronic stress, resilience, and depression in 104 cancer patients. Results indicated that at low to moderate levels of chronic stress, individuals showed higher levels of resilience and lower levels of depression. In relation to early-life adversity, one notable example of longer-term outcomes of trauma and adversity is the recent longitudinal project by Maercker and colleagues. This research examined psychopathological and resilient outcomes in an adult sample of former indentured child laborers in Switzerland (i.e., the so-called *Verdingkinder)*. Many former Verdingkinder experienced high levels of exposure to trauma and maltreatment in childhood, including physical, emotional, and sexual abuse, as well as physical and emotional neglect (1, 12). Findings showed that some former Verdingkinder had developed mental health disorders in later life: 23% major depressive disorder, 26.3% posttraumatic stress disorder, and 7.8% generalized anxiety disorder (13, 14). Nevertheless, despite the experiences of early-life trauma and adversity, results showed that many former Verdingkinder had no mental health disorders and some also showed indications of resilience in later life (15).

Despite the increasing research interest on resilience in the aftermath of stress or adversity, there is a lack of information on the underlying mechanisms which influence the development of this stress-related resilience. The interplay between risk and resilience factors needs to be further explored in order to better understand resilience processes and interindividual differences in psychological health and well-being. One factor which may play an important role in the development of stress-related resilience is the recently revised Sense of Coherence (SOC-R) concept. The SOC-R concept and scale was developed by Bachem and Maercker (16) as a revision of the original Sense of Coherence (SOC), which had shown conflicting results with regard to its psychometric properties (17–19). The original SOC refers to a way of viewing the world which facilitates successful coping with stressors and is comprised of three components: (1) *Comprehensibility*, that stimuli are perceived as structured, predictable, and explicable; (2) *Manageability*, that adequate resources are available to meet demands; and (3) *Meaningfulness*, that demands are viewed as worth investing in and engaging with (20, 21). While the revised SOC-R concept builds on Antonovsky's research, it assumes a more neutral position on the predictability of events and instead focuses on dealing with the ambiguity of life experiences (16).

SOC-R refers to an individual's ability to integrate and balance both positive and negative experiences in order to maintain and develop health and well-being (16). This ability is proposed to develop through the successful coping with and overcoming of experiences of stress or adversity (21). It may therefore be considered an indicator of stress-related resilience aspects. Similar to the original SOC, the SOC-R concept and scale is comprised of three theoretical dimensions and also includes the manageability dimension: (1) *Manageability*, the ability to come to terms and deal with difficult situations. However, two new dimensions were developed to reflect the revised concept: (2) *Reflection*, the ability to consider different perspectives and understand connections, and (3) *Balance*, the ability to balance positive and negative experiences and feelings (16). Regarding the theoretical assumptions of SOC-R, it is suggested that through these three aspects of SOC-R, individuals are able to mobilize available and appropriate resources in order to cope with stressors and adversity. Furthermore, SOC-R is assumed to be relatively stable later in life and a strong SOC-R is assumed to facilitate healthy aging through the maintenance of psychological health and well-being (16, 22).

Initial research with the SOC-R examined its role in overcoming minor adversities (in the form of daily hassles), as well as major adversities (16). Participants included a bereaved sample (*n* = 334) and a control sample from the general population (*n* = 157). Results of this study indicated that SOC-R may be a useful coping mechanism for both minor adversities and extreme stressors. This study also compared the original SOC and SOC-R. With regard to total scores, lower SOC-R was observed in the bereaved sample in comparison to the general population sample. These results were consistent with the findings for the original SOC scale. In addition, construct validity was improved in the SOC-R scale, as SOC-R showed lower correlations than the original SOC with measures of psychological well-being, such as optimism, neuroticism, and self-efficacy (16). These initial findings suggested that the SOC-R scale may be a suitable alternative to the original SOC scale. Further studies have examined the moderating role of SOC-R on the association between adversity health outcomes. One such study examined the influence of SOC-R on the relationship between early-life adversity (in the form of emotional neglect) and mental health in later life (23). Results showed that SOC-R significantly moderated this relationship and acted as a buffer against the negative effects of emotional neglect, with stronger SOC-R associated with better mental health. A more recent study also investigated the interaction between SOC-R and value orientations in predicting posttraumatic growth in bereaved parents (24). Results found a significant interaction between SOC-R and the value self-transcendence, with stronger SOC-R associated with higher levels of posttraumatic growth.

The above research suggests that the strength of an individual's SOC-R can influence their ability to cope with stress or adversity, with stronger SOC-R associated with better outcomes. However, thus far no research exists on the potential mediating role of SOC-R. Previous research with the original SOC construct has shown that SOC can mediate the relationship between adversity and health. For instance, a study with *N* = 193 participants from the general population found that the relationship between adversity (as indicated by worry, anxiety, and stress) and psychological well-being (as indicated by satisfaction with life) was best explained through the significant mediation of SOC (25). Additionally, a more recent longitudinal study examined the mediating role of SOC in *N* = 162 cancer patients. Results showed that following a diagnosis of breast cancer, SOC significantly mediated the change in health-related quality of life over a 6-month period, as indicated by factors such as global quality of life and cognitive, social, and emotional functioning (26). Based on these findings, it is anticipated that SOC-R would also act as a mediator between stress or adversity and indicators of psychological health and well-being. However, the mediating role of SOC-R remains unclear and further research is needed in this area.

Furthermore, as the development of SOC-R is closely linked to the experiences of stress or adversity, the strength and influence of SOC-R may therefore differ depending on the type and severity of the adversity, as well as the stage in the lifespan at which it occurs (23, 27). The majority of the research on SOC-R to-date has focused on early-life adversity and event-specific adversity (e.g., bereavement). Research is therefore needed to examine the role of SOC-R with different types and severities of adversity.

Related to severity is the concept of “steeling.” The steeling effect suggests that previous exposure to some or moderate amounts of stress or adversity can strengthen an individual by increasing their resilience and resistance to later stress (28). In comparison, extreme stress or adversity may be too overwhelming to facilitate successful coping, and minimal stress or adversity may not be sufficiently challenging to necessitate the development of coping abilities. Moderate stress or adversity is therefore proposed to be more beneficial than extreme or even minimal stress or adversity (28, 29). Thus, in accordance with the steeling effect, a non-linear, quadratic (i.e., U-shaped) relationship should exist between stress or adversity and well-being (29). Studies have therefore focused on testing the steeling effect theory by assessing curvilinear (i.e., non-linear) rather than linear models of adversity. Results have demonstrated curvilinear relationships between different types of stress or adversity and indicators of health and well-being. For example, some studies have shown optimal health outcomes at moderate levels of early-life adversity [e.g., 30], lifetime adversity [e.g., 31], and perceived stress [e.g., 32]. In addition, a recent study examined the underlying factors involved in steeling by investigating the relationship between early-life adversity, mental health, and successful aging (33). Findings supported the steeling effect and showed optimal levels of successful aging at moderate levels of early-life adversity and that mental health was a significant mediator of this relationship. These studies provide initial evidence for a steeling effect. However, it is a relatively new and emerging area of resilience research and little is known about the underlying mechanisms which may influence the steeling effect. Given the function of SOC-R in overcoming stress or adversity, it may also play a role in steeling processes. Further research is required to clarify the role of SOC-R in the development of stress-related resilience and, in turn, its influence on health and well-being.

Therefore, to address the gaps identified in the literature, the main aim of the current study was to examine the potential mediating and moderating roles of SOC-R in the relationship between stress or adversity and indicators of psychological health and well-being. As recommended by Fossion et al. (27) and Mc Gee et al. (23), and to build on the existing research with SOC-R, the current study assessed two types of stress or adversity, occurring at different stages in the lifespan: early-life adversity (i.e., childhood trauma and maltreatment) and recent chronic stress. Related to this main aim, two hypotheses were tested: First, based on the theoretical assumptions of SOC-R and the existing empirical evidence, it was hypothesized that SOC-R would significantly moderate the relationship between stress or adversity and indicators of psychological health and well-being (i.e., general mental health, satisfaction with life). It was expected that individuals with a strong SOC-R would show better psychological health than individuals with weaker SOC-R, even with high levels of stress or adversity. Second, it was hypothesized that SOC-R would significantly mediate the relationship between stress or adversity and indicators of psychological health and well-being (i.e., general mental health, satisfaction with life). Finally, an additional aim of this study was to conduct an exploratory analysis to investigate the steeling effect and the role of SOC-R in steeling processes. It was therefore expected that moderate levels of stress or adversity would be associated with stronger SOC-R, which in turn would lead to optimal psychological health and well-being (i.e., general mental health, satisfaction with life).

## Materials and methods

### Study design and procedure

The current study was part of an overarching, longitudinal research project on the steeling effect (“Healthy Aging Against the Odds—Mechanisms behind the Steeling Effect”). A quantitative survey composed of standardized questionnaires was used in this study to assess positive and negative experiences, stress or adversity, current health and well-being, and resilience-related resources. The study was organized and conducted in the University of Zurich and was conducted with the informed consent of all participants in accordance with the Declaration of Helsinki. The protocol was approved by the Swiss ethics committee of the Canton of Zurich (ID 2015-00135) and the Ethics Committee of the Faculty of Arts and Social Sciences in the University of Zurich, Switzerland.

### Participants

Eligible participants were those who met the following inclusion criteria: adults aged 50 years or older, and native Swiss-German speakers. G^*^Power software was initially used to calculate the statistical power analysis. In addition, empirical research recommendations for mediation and moderation analyses were also taken into consideration in determining the required sample size. A minimum sample of *N* = 224 was required in order to detect small to medium effect sizes, with a significance level of *p* = 0.05, and statistical power (1-β) of 0.80 (34–36).

### Measures

#### Sense of coherence scale—revised [SOC-R; 16]

The SOC-R scale assesses an individual's ability to perceive and integrate both positive and negative life experiences in order to maintain and develop health (16). As SOC-R is assumed to develop within the context of adversity, it was used in the current study as an indicator of a stress-related resilience resource. The SOC-R scale is comprised of 13 items rated on a five-point Likert scale and yields a single score. The three dimensions which comprise the scale are: *Manageability* (e.g., “One can always find a way to cope with painful things in life”), *Balance* (e.g., “In my thoughts and actions I take into account that things often have two sides: good and bad ones”), and *Reflection* (e.g., “Normally I can consider a situation from various perspectives”). It is available in German and English, and results from the German version show high internal consistency of between α = 0.75 and 0.81 for the total scale, and sufficient internal consistency of between α = 0.54 and 0.77 for the subscales (16, 23). The SOC-R scale has also been shown to have high test-retest reliability, with an *r* = 0.85 over a 1-month period, and *r* = 0.74 over an interval of 15 months (16).

#### Childhood trauma questionnaire [CTQ; 37]

The CTQ measures trauma and adversity experienced early in life. It is comprised of 28 items rated on a five-point Likert scale. It is composed of the following five subscales, with each subscale assessed by 5 items: emotional abuse, physical abuse, sexual abuse, emotional neglect, and physical neglect. It also includes a three-item minimization-denial scale to detect false-negative trauma reports. The German version shows sufficient internal consistency across all subscales, with a Cronbach's alpha of between α = 0.55 and 0.96 (38, 39).

#### Screening scale of chronic stress [SSCS; 40]

The SSCS is a screening subscale of the Trier Inventory for the Assessment of Chronic Stress (TICS) and measures perceived stress over the previous 3-month period. It assesses five domains of stress: chronic worrying, work-related overload, social overload, excessive demands, and lack of social recognition. It consists of 12 items rated on a five-point Likert scale and yields a single score. The German version shows high internal consistency of α = 0.87 (41).

#### 36-item short form health survey version 2 [SF-36 V2; 42, 43]

The SF-36 measures current mental and physical health. It consists of 36 items and is comprised of eight subscales which combine to form the two distinct component summary scores for mental and physical health. The physical component consists of four subscales: physical functioning, role-physical, bodily pain, and general health; and the mental component consists of four subscales: vitality, social functioning, role-emotional, and mental health (43). To calculate the component summary scores, population- and gender-specific norms for means, standard deviations, and factors loadings are used. As country-specific norms are currently unavailable for Switzerland, German norms were used in the current study (44, 45). The mental health component was used in the current study as an indicator of psychological health. The German version shows high internal consistency across the eight subscales, with a Cronbach's alpha of between α = 0.81 and 0.94 (43).

#### Satisfaction with life scale [SWLS; 46]

The SWLS measures subjective well-being in relation to global life satisfaction (47). It consists of five items rated on a seven-point Likert scale and yields a single score. The German version shows high internal consistency of α = 0.92 (48).

### Procedure

A longitudinal study was conducted in the German-speaking regions of Switzerland. The study consisted of two assessment time points: baseline assessment at T1 (summer 2016) and a follow-up assessment 12 months later at T2 (summer 2017). Study participants were recruited using advertisements on websites, in newspapers and magazines, posted flyers, and radio interviews with the authors. Participants were also recruited through the University Research Priority Program “Dynamics of Healthy Aging” in the University of Zurich. The survey was available as an online survey or in pen-and-paper format. Individuals who were interested in taking part contacted the research team and were either emailed a link to the online survey or were posted a pen-and-paper survey package. A study incentive was provided at each assessment point, with participants who completed the survey being entered into a raffle for 10 shopping vouchers.

The online survey was programmed using *Unipark* software (49). After following the link to the online survey, participants were provided with the study information sheet and then the informed consent form. Participants provided informed consent online by ticking the corresponding box to indicate their consent to participate in the study. Only if they provided informed consent could participants go on to complete the questionnaires. The pen-and-paper survey package also contained an information sheet, an informed consent form, and the questionnaire survey, as well as a free-post return envelope. At T2, participants were provided with either the online survey link or the pen-and-paper survey package depending on their preference at T1. Both survey formats were randomized at the scale level for each participant in order to avoid sequence and order effects. All assessment instruments were repeated at T2, except for the CTQ. As the CTQ assessed trauma and adversity experienced in childhood, this data was collected at baseline (T1) and the scale was then removed from the survey at T2 to reduce participant burden. Only data for which informed consent had been provided was included in the dataset and analyses.

### Statistical analysis

IBM Statistical Package for Social Sciences (SPSS) version 25.0 and PROCESS version 3.0 macro for SPSS were used to analyze the data (50, 51). For each instrument, less than 1% missing values were observed. Little's missing completely at random (MCAR) test suggested that most of the values were MCAR and were therefore replaced using the Expectation-Maximization algorithm (52, 53). For values not missing at random, the means on the subscale level were calculated for each participant.

The mediating and moderating roles of SOC-R were examined in the relationship between past and recent adversity and psychological health and well-being. According to the conceptual framework for longitudinal research proposed by Collins (54), the theoretical model of the anticipated change should be considered in the operationalization of the statistical model. Regarding the current study, according to the theoretical assumptions of SOC-R, SOC-R should be relatively stable later in life (16, 20). Therefore, as the current sample consisted mainly of adults and older adults, it was anticipated that SOC-R would not change significantly over the two assessment points. A paired-samples *t*-test was conducted to test this assumption. In line with the theoretical assumption, the results showed that SOC-R scores did not differ significantly [*t*_(237)_ = 1.61, *p* = 0.11; *r* = 0.10] at T1 (*M* = 50.61, *SD* = 5.72) and T2 (*M* = 50.12, *SD* = 5.62). Therefore, baseline SOC-R was used for the longitudinal mediation and moderation analyses with the CTQ subscales. In order to examine the influence of more recent stress, T2 variables were assessed with chronic stress as the predictor.

Mediation analyses (model 4) and moderation analyses (model 1) were conducted using the PROCESS version 3.0 macro for SPSS (50). Socio-demographic variables (i.e., age and education) which showed significant correlations with the predictor and outcomes variables were included as covariates in these analyses to control for potential confounding. Where effects were not observed for total SOC-R, analyses were conducted with the SOC-R subscale level in order to probe potential underlying effects from the dimensions which comprise and ultimately influence SOC-R. Regarding the moderation analyses, a “regions of significance” analysis was also conducted using the Johnson-Neyman procedure (55). This procedure provides additional information on the significance regions for the effect of the predictor (early-life adversity and chronic stress) on the outcome (general mental health and satisfaction with life) at specific values of a continuous moderator (SOC-R). In addition, in order to investigate the role of SOC-R in steeling processes curvilinear (i.e., non-linear) associations, specifically quadratic relationships, must be assessed. To examine these potential curvilinear relationships, the linear stress and adversity terms must first be controlled for by including these variables as covariates in the model. The quadratic (i.e., squared) terms for early-life adversity (CTQ^2^) and chronic stress (SSCS^2^) were then implemented into the mediation model (29, 56). Within this model, a steeling effect is indicated by a significant relationship between the quadratic adversity and stress terms and the mediator (i.e., SOC-R), and with the health and well-being indicators of general mental health and satisfaction with life.

## Results

### Sample characteristics

A total of 337 participants were recruited at T1. From this, 260 participants completed both assessments (T1 and T2) and 77 participants dropped out after T1. Participants who completed both T1 and T2 assessments showed a higher SOC-R score (*M* = 50.5, *SD* = 5.79), than those who dropped out after T1 (*M* = 48.6, *SD* = 6.93). An independent samples *t*-test showed that this difference, 1.93, BCa 95% CI [0.217, 3.64], was significant *t*_(328)_ = 2.44, *p* = 0.03, with a small effect size (*r* = 0.13). From the 260 participants at T2, *n* = 17 participants were excluded from the analyses due to data missing at the total scale or subscale level and a further *n* = 5 multivariate outliers were removed. The final sample consisted of *N* = 238 participants, with a mean age of 68.31 years (*SD* = 8.96, age range = 50–92 years). The sample was comprised of 175 females (73.5%) and 63 males (26.5%). The online survey was completed by 160 participants (67.2%, *M*_age_ = 65.94 years, *SD* = 8.27) and the pen-and-paper survey was completed by 78 participants (32.8%, *M*_age_ = 73.17 years, *SD* = 8.39). The majority of participants indicated that vocational training (33.2%) was their highest level of education, followed by university-level education at university of applied sciences (18.5%) and university (16.0%). Regarding employment status, 110 participants (46.2%) were retired, 65 (27.3%) were employed, 38 (16.0%) were involved in voluntary activities, and 7 (2.9%) were unemployed. Regarding relationship status, the majority of participants were married (35.3%), followed by widowed (16.4%), and divorced (15.5%). See Table 1 for an overview of the sample characteristics. With regard to early-life adversity, participants reported higher levels of emotional neglect (*M* = 13.60; *SD* = 5.55) and emotional abuse (*M* = 10.43; *SD* = 5.49), followed by physical neglect (*M* = 8.41; *SD* = 3.12), physical abuse (*M* = 7.15; *SD* = 3.43), and sexual abuse (*M* = 7.08; *SD* = 3.76). However, levels of experienced early-life adversity were low for most categories, with a high percentage of participants reporting none to low levels of adversity: sexual abuse (73.9%, *n* = 176), physical abuse (84.5%, *n* = 201), physical neglect (71.4%, *n* = 170), emotional abuse (70.2%, *n* = 167), and emotional neglect (59.7%, *n* = 142).

**Table 1:** Sample characteristics.

**Sample Characteristics**	**Total (*****N*** = **238)**		**Male**		**Female**	
	**M**	***SD***	***n***	***%***	***n***	***%***
Age (years; age range = 50–92 years)	68.31	8.96	–	–	–	–
	***n***	**%**				
Gender (% female)	175	73.5	–	–	–	–
**Education level:**						
Primary school	5	2.1	1	1.6	4	2.3
Secondary/High school	18	7.6	4	6.3	14	8.0
Vocational training	79	33.2	18	28.6	61	34.9
Specialized vocational training	36	15.1	8	12.7	28	16.0
University of Applied Sciences	44	18.5	13	20.6	31	17.7
University	38	16.0	14	22.2	24	13.7
Other	18	7.6	5	7.9	13	7.4
**Employment status: (*****n*** = **228)**						
Employed	65	27.3	14	22.2	51	29.1
Unemployed	7	2.9	3	4.8	4	2.3
Voluntary work	38	16.0	4	6.3	34	19.4
Retired	110	46.2	40	63.5	70	40.0
Other	8	3.4	1	1.6	7	4.0
**Marital status: (*****n*** = **231)**						
Single	36	15.1	7	11.1	29	16.6
Committed relationship	29	12.2	8	12.7	21	12.0
Married	84	35.3	32	50.8	52	29.7
Separated	6	2.5	–	–	6	3.4
Divorced	37	15.5	8	12.7	29	16.6
Widowed	39	16.4	5	7.9	34	19.4

### Moderation analysis

Moderation analyses were conducted to examine the influence of SOC-R on the strength of the relationship between different indicators of stress or adversity (i.e., early-life adversity, chronic stress) and current psychological health and well-being (i.e., general mental health, satisfaction with life). With regard to early-life adversity, no significant interaction effects were observed at the SOC-R total or subscale level for the CTQ subscales (physical, sexual, emotional abuse, and physical or emotional neglect).

Regarding chronic stress, no significant interaction effects were shown for total SOC-R. However, on the subscale level, a significant interaction effect was observed for general mental health and the manageability subscale of SOC-R (*b* = 0.04, 95% CI [0.007, 0.082], *t* = 2.32, *p* < 0.05). This suggests that the manageability dimension of SOC-R significantly moderates the relationship between chronic stress and general mental health (see Table 2 for the predictors of general mental health and the interaction effect).

**Table 2:** Predictors of general mental health and the significant interaction effect, with SOC-R Manageability as the moderator.

**Predictor**	***b* [CI]**	***SE B***	***t***	***p***
Constant	51.98 [44.88, 59.07]	3.60	14.44	0.000
SOC-R Manageability (centered)	0.912 [0.525, 1.300]	0.197	4.64	0.000
Chronic stress (centered)	−0.603 [−0.734, −0.473]	0.066	−9.10	0.000
SOC-R Manageability x Chronic stress	0.044 [0.007, 0.082]	0.019	2.32	0.021

*R^2^ = 0.46; b = unstandardized beta; SE B = standard error for the unstandardized beta; t = t test statistic; p = p-value*.

**Figure 1:**
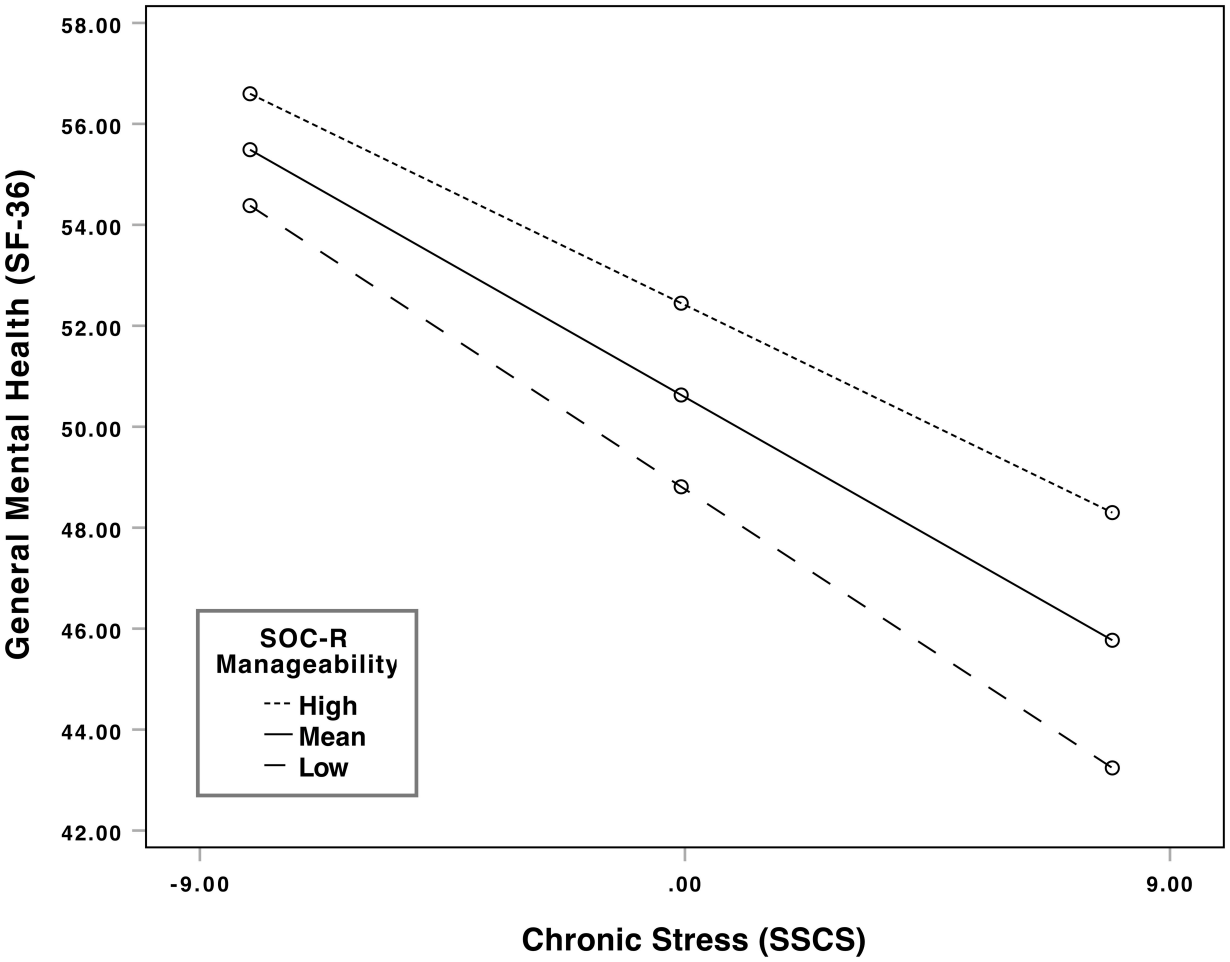
Significant interaction between chronic stress and general mental health at different levels of the moderator (SOC-R manageability). High and low levels refer to one standard deviation above and below the mean of the moderator (i.e., SOC-R Manageability).

Additionally, a significant negative relationship was observed between chronic stress and general mental health at low (*b* = −0.696, 95% CI [−0.842, −0.551], *t* = −9.43, *p* < 0.01); mean (*b* = −0.607, 95% CI [−0.738, −0.477], *t* = −9.19, *p* < 0.01); and high levels of manageability (*b* = −0.519, 95% CI [−0.674, −0.363], *t* = −6.58, *p* < 0.01). High and low levels refer to one standard deviation above and below the mean of the moderator (i.e., SOC-R Manageability). However, the decrease in effect (i.e., *b*-values) suggested a buffering effect of manageability. Supporting this, results of the Johnson-Neyman procedure showed that as manageability increased, the strength of the relationship between chronic stress and general mental health changed from a strong negative effect (*b* = −0.963) to a small negative effect (*b* = −0.341). This indicates that the stronger the manageability, the weaker is the negative effect of chronic stress on general mental health. Visual inspection of the interaction plot also indicated a buffering effect. Individuals with a higher level of manageability had higher scores of general mental health across all levels of chronic stress in comparison to individuals with mean and low levels of manageability. This difference was particularly evident when levels of chronic stress were high. See Figure 1 for the graph of the significant moderation and interaction effects.

### Mediation analysis

Mediation analyses investigated whether SOC-R acts as a mediator of the relationship between stress or adversity (i.e., early-life adversity, chronic stress) and current psychological health and well-being (i.e., general mental health, satisfaction with life). With regard to early-life adversity, a significant indirect effect was observed for the CTQ subscales emotional neglect and physical neglect, for both general mental health and satisfaction with life. No significant indirect effects were observed for the abuse subscales of the CTQ (emotional, physical, sexual abuse). With regard to chronic stress, a significant indirect effect was also observed for both general mental health and satisfaction with life.

#### CTQ: emotional neglect

**Figure 2:**
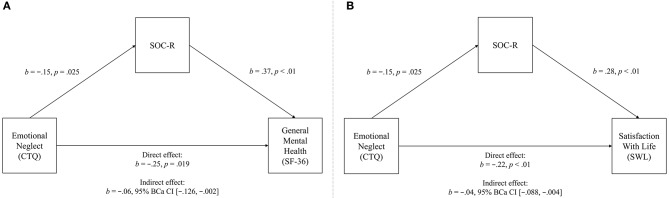
Mediation model of the relationships between emotional neglect (predictor) and general mental health (outcome variable, **A**), and satisfaction with life (outcome variable, **B**), significantly mediated by SOC-R (mediator).

Regarding emotional neglect, a significant negative total effect was initially observed (*b* = −0.309, 95% CI [−0.524, −0.094], *t* = −2.83, *p* < 0.01), explaining 4.23% of the variance in general mental health. However, when SOC-R was included as a mediator in the model, a significant direct effect emerged, explaining a greater percentage of the variance (9.11%). A significant indirect effect was also observed, indicating that SOC-R significantly mediated the relationship between emotional neglect and general mental health. See Figure 2A for the full mediation model.

Similarly, a significant negative total effect was initially observed for satisfaction with life (*b* = −0.259, 95% CI [−0.388, −0.129], *t* = −3.93, *p* < 0.01), explaining 8.67% of the variance. However, when SOC-R was included as a mediator in the model, a significant direct effect emerged, explaining a greater percentage of the variance (16.13%). A significant indirect effect was also observed, indicating that SOC-R significantly mediated the relationship between emotional neglect and satisfaction with life. See Figure 2B for the full mediation model.

#### CTQ: physical neglect

**Figure 3:**
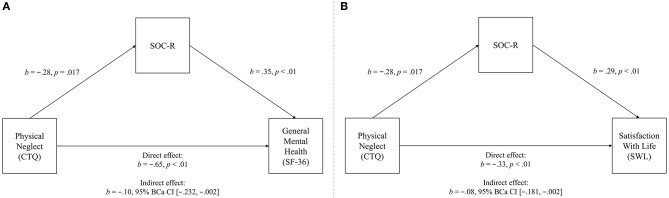
Mediation model of the relationships between physical neglect (predictor) and general mental health (outcome variable, **A**), and satisfaction with life (outcome variable, **B**), significantly mediated by SOC-R (mediator).

Regarding physical neglect, a significant negative total effect was initially observed (*b* = −0.746, 95% CI [−1.125, −0.367], *t* = −3.88, *p* < 0.01), explaining 6.92% of the variance in general mental health. However, when SOC-R was included as a mediator in the model, a significant direct effect emerged, explaining a greater percentage of the variance (11.32%). A significant indirect effect was also observed, indicating that SOC-R significantly mediated the relationship between physical neglect and general mental health. See Figure 3A for the full mediation model.

In addition, a significant negative total effect was initially observed for satisfaction with life (*b* = −0.407, 95% CI [−0.640, −0.173], *t* = −3.43, *p* < 0.01), explaining 7.31% of the variance. However, when SOC-R was included as a mediator in the model, a significant direct effect emerged, explaining a greater percentage of the variance (14.93%). A significant indirect effect was also observed, indicating that SOC-R significantly mediated the relationship between physical neglect and satisfaction with life. See Figure 3B for the full mediation model.

#### SSCS: chronic stress

**Figure 4:**
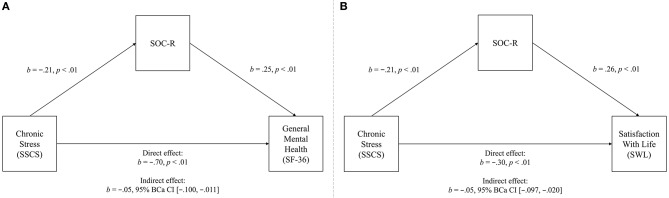
Mediation model of the relationships between chronic stress (predictor) and general mental health (outcome variable, **A**), and satisfaction with life (outcome variable, **B**), significantly mediated by SOC-R (mediator).

Regarding chronic stress, a significant negative total effect was initially observed (*b* = −0.756, 95% CI [−0.880, −0.632], *t* = −12.04, *p* < 0.01), explaining 39.38% of the variance in general mental health. However, when SOC-R was included as a mediator in the model, a significant direct effect emerged, explaining a greater percentage of the variance (41.45%). A significant indirect effect was also observed, indicating that SOC-R significantly mediated the relationship between chronic stress and general mental health. See Figure 4A for the full mediation model.

A significant negative total effect was also initially observed for satisfaction with life (*b* = −0.359, 95% CI [−0.443, −0.275], *t* = −8.38, *p* < 0.01), explaining 26.01% of the variance. However, when SOC-R was included as a mediator in the model, a significant direct effect emerged, explaining a greater percentage of the variance (31.93%). A significant indirect effect was also observed, indicating that SOC-R significantly mediated the relationship between chronic stress and satisfaction with life. See Figure 4B for the full mediation model.

### Steeling effect

Additionally, to examine the role of SOC-R in steeling processes, the quadratic terms for early-life adversity (CTQ^2^) and chronic stress (SSCS^2^) were implemented in the mediation model to assess potential curvilinear relationships (29). To detect evidence of steeling, a curvilinear relationship should exist between adversity and stress and SOC-R, and between adversity and stress and the health and well-being outcomes (i.e., general mental health, satisfaction with life). However, results showed no significant indirect effects through SOC-R, and no significant quadratic relationships were observed between adversity and stress and SOC-R. This suggests that highest level of SOC-R was not shown in participants with moderate levels of adversity or stress, and that SOC-R did not significantly mediate the curvilinear relationship between adversity and stress and the indicators of psychological health and well-being.

## Discussion

The purpose of the current study was to examine the moderating and mediating roles of SOC-R in the development of stress-related resilience, and to investigate its potential role in steeling processes. Results showed that the Manageability subscale of SOC-R significantly moderated the relationship between chronic stress and psychological health. Results further showed that SOC-R was a significant mediator of the relationship between certain early-life adversities (i.e., physical and emotional neglect), as well as chronic stress, and psychological health and well-being. Finally, results revealed no curvilinear relationships between adversity or stress and SOC-R, indicating no evidence for a steeling effect.

Regarding moderation, total SOC-R was not shown to moderate the relationship between early-life adversity and health or well-being. This finding is in contrast to previous research, which examined the moderating role of SOC-R in a Swiss sample (23). Findings showed that total SOC-R moderated the relationship between emotional neglect in childhood and current mental health (23). One explanation may be that the sample in the current study was somewhat older than in the previous study by Mc Gee et al. (23). It may be that with increasing age, the focus of health-related resources, such as SOC-R, shifts toward more immediate health concerns such as physical or functional health (57). In relation to the original SOC, studies often found a weaker relationship with physical health in comparison with mental health (58). For example, recent research by Gison et al. (59) examined the predictive effect of SOC on psychological and physical outcomes in participants with Parkinson's Disease. Results showed that SOC was predictive of health-related quality of life and emotional distress, but not physical disability. However, the influence of SOC-R on physical health has not yet been assessed and future research is required to further examine this relationship across various age groups with a diversity of physical health statuses.

SOC-R (total) was also not found to moderate chronic stress and health or well-being. However, the Manageability subscale was shown to be a significant moderator. This would tentatively suggest partial support for the first hypothesis (i.e., that SOC-R would significantly moderate the relationship between stress or adversity and indicators of psychological health and well-being). Individuals with strong Manageability showed better general mental health scores than individuals with weaker Manageability, even when levels of chronic stress were high. Furthermore, similar to the moderation results of the study by Mc Gee et al. (23), a buffering effect was observed so that as Manageability increased, the negative effect of chronic stress on general mental health decreased. The Manageability dimension of SOC-R refers to the ability to come to terms and deal with difficult situations (16). These findings may therefore indicate that the ability to manage stress over prolonged periods is an important aspect of successful coping, over and above the influence of the Reflection or Balance dimensions. In support of this, the importance of Manageability for coping was highlighted in the initial evaluation study of SOC-R by Bachem and Maercker (16). Results found that the Manageability dimension explained the largest proportion of variance in the bereaved sample. Bereavement is a major adversity, which would (similar to a chronic stressor) require long-term coping management abilities.

With regard to mediation, SOC-R (total) was shown to be a significant mediator for some types of early-life adversity and chronic stress. While no significant mediations were observed with the abuse subscales (of early-life adversity), this is not entirely unexpected, given the low number of reported physical and sexual abuse in the general population sample. In line with previous general population studies in Germany [see Glaesmer (60)], experiences of neglect were more prevalent in the current study than experiences of abuse. However, in support of the second hypotheses (i.e., that SOC-R would significantly mediate the relationship between stress or adversity and indicators of psychological health and well-being), the results suggest that SOC-R was a significant mediator for general mental health and satisfaction with life, in relation to childhood physical neglect, childhood emotional neglect, and recent chronic stress. Although no previous research exists on the mediating role of SOC-R, findings are consistent with and expand upon the empirical research indicating a mediating influence with the original SOC (58, 61). The finding that SOC-R may explain the relationship between stress or adversity and psychological health and well-being also supports the assumption that SOC-R may be an integral mechanism underpinning the development of stress-related resilience.

In contrast to expectations, results did not support a steeling effect (i.e., that moderate levels of stress or adversity would be associated with stronger SOC-R, which in turn would lead to optimal psychological health and well-being). This is also inconsistent with recent research, which found significant curvilinear relationships between early-life adversity and quality of life outcomes, including general mental health (33). This may suggest that moderate levels of adversity are not “optimal” for the development of a strong SOC-R. However, another explanation may be due to differences in the adversity indicator, as the operationalization of early-life adversity in the study by Höltge et al. (33) differed to that in the current study. Nevertheless, before definitive conclusions can be drawn, further research is required to assess SOC-R and the steeling effect in larger, representative samples, with a greater range of adversity. Although a steeling effect was also not observed for chronic stress, this was consistent with previous research. Similar to the current study, research by Dooley et al. (62) found evidence of a linear relationship (i.e., as in the mediation analysis of the current study) but not a curvilinear (steeling) relationship between chronic stress and well-being. One explanation may be that prolonged stress experiences have often been shown to lead to increased sensitization to later stress rather than an increased resistance to later stress (4, 7, 28. Nevertheless, these findings highlight the importance of considering the type and severity of the stressor and adversity in the interplay between risk and resilience factors, and the resulting resilience or psychopathology (23, 27).

### Limitations and future directions

Directions for future research can be identified by addressing some limitations of the current study. First, the retrospective nature of the study design may have led to recall bias, particularly in relation to the more distant experiences of early-life adversity (63). Similarly, the use of self-report assessments may also have led to biased reporting. To more accurately capture the influence of stress or adversity exposure, future research should use prospective, longitudinal designs, and include objective measures of stress or adversity, such as cortisol activity in response to stress-tests [e.g., 32]. Another limitation of this study was the low levels of adversity, particularly early-life adversity, in the current sample. For instance, in relation to early-life adversity, the large-scale, cross-national study by Kessler et al. (3) found that in a sample of *n* = 20,652 participants from high-income countries (from the same World Bank classification as Switzerland), 38.4% reported having experienced childhood adversity. Furthermore, in relation to childhood sexual abuse, a recent nationally-representative survey of *N* = 6,787 adolescents was conducted in Switzerland (64). Results showed that 40.2% (*n* = 1,282) of girls and 17.2% (*n* = 610) of boys reported having experienced at least one form of child sexual abuse. While the prevalence of adversities was generally lower in the current study, the highest reported adversities were emotional and physical neglect. However, this may be expected in a general population sample and the lower levels of adversity in the other categories may explain the lack of significant results. Related to this is self-selection bias, which may have influenced the composition of the sample, as individuals who experienced less adversity may be more likely to choose to participate in the study (65). Similarly, differences observed in SOC-R between participants and drop-outs may indicate a selection bias. Those who dropped out after T1 showed a significantly lower SOC-R score than those who completed both assessment points. It may be that individuals with a higher SOC-R are more likely to initially volunteer to take part in a study and to persevere with it at T2. To improve confidence in the findings, future research should replicate these analyses using different sampling techniques in both clinical and non-clinical samples with a greater range of SOC-R, as well as stressor and adversity severities.

Another limitation is the relatively small sample size, which may restrict the generalization of results to the general population. Nevertheless, research from large-scale studies on resilience support the finding that resilience can buffer the negative effects of stress or adversity. For instance, a recent study with a representative German community sample (*N* = 2,508) found that resilient coping was not only associated with lower levels of distress, but also buffered the negative effects of childhood adversities on distress (66). Comparable results were shown in the population-based, longitudinal study: the Virginia Adult Twin Studies of Psychiatric and Substance Use Disorders. Results from *N* = 7,463 participants showed that high levels of resilience at baseline buffered against the development of psychopathology, even in the presence of high levels of stressful life events (67). Similarly, a longitudinal Swedish cohort study of *N* = 237,879 participants found that participants with lower stress resilience to chronic stress showed an increased risk of stroke (68). However, SOC-R has yet to be assessed as an indicator of stress-related resilience aspects on such a large scale. Therefore, to increase confidence in the results, it is recommended that future research assess the buffering effect of SOC-R with a large sample size. Furthermore, another sample-related limitation is the broad age range (50–92 years) used in the current study, which may have masked specific age group effects or patterns in SOC-R (69). Future studies could also examine SOC-R across different age groups.

In addition, studies should investigate the different types of adversity (e.g., acute versus chronic stress, cumulative lifetime adversity, event-specific adversities), which may influence the strength of SOC-R and in turn, its impact on health and well-being (16, 23). Finally, the current study investigated the role of SOC-R in steeling processes using a simple mediation model of curvilinear associations. However, SOC-R is assumed to promote the development of stress-related resilience through the appropriate use of resources (16, 22). Future studies may therefore benefit from including resources into the model with SOC-R, such as in the form of moderated mediation (29). This is supported by a review of studies on resilience in stress-related disorders (70). Thirteen studies were included which investigated predictors of resilient outcomes following stress exposure. Results indicated that resilience is a dynamic process, involving the interaction of multiple separate resilience-related factors (70). Similarly, in relation to resilience in older adults, research has shown that that a combination of mental, social, and physical factors is important for resilience. In particular, a recent review identified optimism, adaptive coping, positive emotions, social support, and social connectedness as important factors in the maintenance of high levels of resilience in older adulthood (71). In relation to future moderation and mediation analyses with SOC-R, as indication for which resources to include, studies can also draw on previous research by Mc Gee et al. (23), which examined convergent and discriminant correlations between SOC-R and related psychological concepts. Similar to the recommendations by MacLeod et al. (71), results showed moderate to strong correlations between SOC-R and a number of resources, including general self-efficacy, social support, and optimism.

## Conclusion

By investigating the moderating and mediating roles of SOC-R, the current study provides a meaningful contribution to the research on stress-related resilience. It addresses a gap in the literature, as it is the first study to examine the mediating role of SOC-R in the relationship between stress or adversity and health and well-being. It also builds on previous studies by assessing chronic stress, as well as early-life adversity. While evidence of a steeling effect was not observed, the results suggest that SOC-R may still be a crucial underlying mechanism in the development of resilience. In conclusion, the findings suggest that in overcoming stress or adversity, an individual's SOC-R (and the strength of their SOC-R) plays an important role in fostering resilience and in turn, psychological health and well-being.

## Author contributions

All authors made substantial contributions to the conception or design of the work; or the acquisition, analysis, or interpretation of data for the work; and drafting the work or revising it critically for important intellectual content; and final approval of the version to be published; and agreement to be accountable for all aspects of the work in ensuring that questions related to the accuracy or integrity of any part of the work are appropriately investigated and resolved.

### Conflict of interest statement

The authors declare that the research was conducted in the absence of any commercial or financial relationships that could be construed as a potential conflict of interest. The handling Editor declared a shared affiliation, though no other collaboration, with the authors.
